# Raman Spectroscopy Cell-based Biosensors

**Published:** 2007-07-26

**Authors:** Ioan Notingher

**Affiliations:** University of Nottingham, School of Physics and Astronomy, University Park, Nottingham NG7 2RD, UK.; E-mail: ioan.notingher@nottingham.ac.uk

**Keywords:** Raman spectroscopy, cell-based, biosensors, cells, toxic agents, toxicology

## Abstract

One of the main challenges faced by biodetection systems is the ability to detect and identify a large range of toxins at low concentrations and in short times. Cell-based biosensors rely on detecting changes in cell behaviour, metabolism, or induction of cell death following exposure of live cells to toxic agents. Raman spectroscopy is a powerful technique for studying cellular biochemistry. Different toxic chemicals have different effects on living cells and induce different time-dependent biochemical changes related to cell death mechanisms. Cellular changes start with membrane receptor signalling leading to cytoplasmic shrinkage and nuclear fragmentation. The potential advantage of Raman spectroscopy cell-based systems is that they are not engineered to respond specifically to a single toxic agent but are free to react to many biologically active compounds. Raman spectroscopy biosensors can also provide additional information from the time-dependent changes of cellular biochemistry. Since no cell labelling or staining is required, the specific time dependent biochemical changes in the living cells can be used for the identification and quantification of the toxic agents. Thus, detection of biochemical changes of cells by Raman spectroscopy could overcome the limitations of other biosensor techniques, with respect to detection and discrimination of a large range of toxic agents. Further developments of this technique may also include integration of cellular microarrays for high throughput *in vitro* toxicological testing of pharmaceuticals and *in situ* monitoring of the growth of engineered tissues.

## 1. Introduction

In response to the increasing demand for detection of chemical and biological toxic agents, such as environmental pollutants and warfare agents, various optical techniques have been proposed. Much effort has been focused on developing biosensors that have the potential to respond to a diverse range of toxic chemicals and biological toxins at relevant concentrations and in real-time. The use of living cells as the sensor elements in such systems is one approach that has undergone significant developments during the last decade. In these sensors, the biological reaction of cells is measured following the exposure of single cells, layers of confluent cells or networks and arrays of living cells to toxic agents [1-3]. This approach offers key advantages compared to other biosensing methods, such as molecular and antibody- biosensors: cell-based biosensors are not engineered to respond specifically to a single toxic agent but are free to react to many biologically active compounds.

Many techniques have been proposed for interrogation and measurement of biological response of cellular sensors. Changes in cell behaviour, cell-cell and cell-substrate contact, metabolism, or induction of cell death following exposure of cells to toxic agents can be measured and used to detect the presence and also identify the toxic agents. Optical methods offer a significant advantage as light can be used non-invasively to probe cells. Variations in the intensity of the intrinsic light emitted by bioluminescent bacteria can be used to detect toxic chemicals such as polycyclic aromatic hydrocarbons and phenols [4, 5]. Various fluorescent labels have been developed to monitor cellular functions and a large number of techniques have been implemented for high-throughput screening [6]. Neurotoxic chemicals can be detected by monitoring active neuronal networks using microelectrode arrays [7-11]. Changes in the spontaneous electric activity (e.g. burst duration, frequency) can be measured when neurons are exposed to various neuroactive drugs and toxins [10, 11]. Similar methods using cardiac myocytes have also been reported for studying pesticide [12] and metal toxicity [13]. The extracellular potential of other electrically active cells, such as osteoblasts, can also be measured and related to presence of various toxic agents [14]. Measurement of pH using microelectronic sensors to monitor the acidity of culture media in the vicinity of living cells has also been used to monitor the behaviour of living cells [15]. Cells exposed to toxins known to inhibit membrane ionic pumps show small decreases in the pH which can be detected using integrated microelectronics [15]. Changes in the pH caused by exposure of bacteria engineered genetically to express various enzymes have also been used for the detection of organophosphate toxins, such as nerve agents [16]. Confluent layers of endothelial cells grown on ion-selective membranes were able to detect various concentrations of histamine in the culture medium due to increased permeability caused by cell-cell contact alteration and formation of gaps [17]. Impedance measurements of cellular arrays have been used to determine the behaviour of various cell types, such as fibroblasts [18] and endothelial cells [19].

The main challenge faced by all biosensing techniques is the ability to discriminate between diverse toxic agents. Although the information regarding general toxicity may be useful for classification of a potential agent, identification and quantification are also desirable. With the possible exception of the neuronal network type systems, it has proven difficult to identify and quantify different toxic agents with many of the approaches described. The main reason is that the interrogation of the cellular component results in readout with limited biological information and thus results in similar changes in the detected signal for many different toxic agents. Additionally, many of these techniques require invasive methods of cell sampling, complex cellular modifications or use cell types that are difficult to establish in reliable cell-lines. Such methods limit considerably the time-dependent biological responses of cells, which often are indicative of the type and dose of the toxic agent.

Raman spectroscopy is a powerful analytical technique which has been widely used in the study of biological samples during the last two decades [20-22]. Raman micro-spectroscopy can provide useful biochemical information regarding live cells, which can be related to the interaction with toxic agents or drugs, disease, cell death and differentiation [23]. In addition to conventional non-resonant Raman spectroscopy, there have been numerous reports on using resonant Raman (RS) spectroscopy [24], surface enhanced Raman spectroscopy (SERS) [25] and coherent anti-Stokes Raman spectroscopy (CARS) [26] for studying live cells. These methods are based on various effects to enhance the Raman signal of specific molecules found in cells [23]. These techniques have been widely used for developing sensors and biosensors [27].

The potential of non-resonant conventional Raman spectroscopy as an interrogation method of cells is based on the fact that different toxic chemicals have different effects on living cells and induce specific time-dependent biochemical changes related to cell death mechanisms. The Raman spectrum of a cell represents an information rich “fingerprint” of the overall biochemical composition of the cell, thus different toxic agents that initiate different cellular responses and biochemical changes should produce distinct changes in the Raman spectra. In addition, time-course Raman spectra can be acquired from live cells maintained in physiological conditions and without need of invasive procedures. The information obtained is related to the intrinsic molecular composition of the cell, thus no labels or other contrast enhancing chemicals need to be used. The detection of time-dependent biochemical changes of cells has the potential to provide the additional level of information needed for quantification and discrimination of a wider range of toxic agents. The current article emphasizes the potential of conventional non-resonant Raman spectroscopy as a powerful interrogation method for cell-based biosensors.

## 2. Raman spectroscopy and instrumentation

### 2.1. Raman scattering

**Figure 1.**
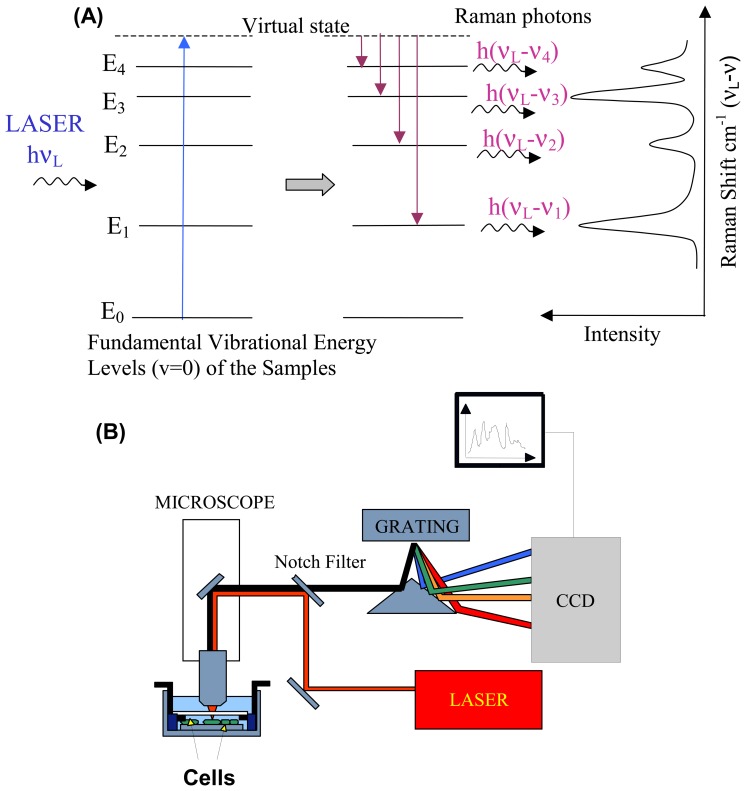
Schematic representation of (A) Raman Stokes scattering of laser photons by vibrating molecules in the sample: energy is transferred by laser photons to the molecules as vibrational energy, the energy loss correspond to the vibrational energy levels of the molecules (E1, E2, …); and (B) experimental set-up for Raman spectroscopy measurements of cells.

When photons are incident on a sample, they can be scattered elastically (no change in energy) or inelastically (change in energy) by the sample. Raman spectroscopy is based on inelastic scattering of photons following their interaction with vibrating molecules of the sample. During this interaction, photons transfer (Stokes)/receive (Anti-Stokes) energy to/from molecules as vibrational energy. Thus, the energy change of the scattered photons corresponds to the vibrational energy levels of the sample molecules (Figure 1A). In Figure 1A, the energy levels E_1_, E_2_, E_3_ and E_4_ represent the fundamental vibrational levels (vibrational quantum number v=0) corresponding to normal vibrational modes of the molecule of frequencies ν_1_, ν_2_, ν_3_ and ν_4_. Since the vibrational energy spectrum depends on the chemical composition of the sample (type of atoms, bond strength, bond angles, symmetry, etc), a Raman spectrum has an unrivalled chemical specificity and represents a chemical fingerprint of the sample. For more detailed description of the physics of the Raman effect see references [28-30].

### 2.2. Instrumentation

Raman scattering is normally excited using a laser in the visible or near-infrared region and the energy (frequency or wavenumber) spectrum of the scattered photons is analysed using a dispersion spectrometer (Figure 1B). A diffraction grating is used to disperse the Raman scattered beam into multiple beams corresponding to specific frequencies which are subsequently focused on an array of detectors, such as a high sensitivity charge coupled device (CCD). The spectrometer has to be equipped with a notch or edge filter to reject the elastically scattered photons (Rayleigh photons), which have the same energy as the laser photons, which would produce an intense background covering the Raman photons.

In Raman micro-spectroscopy, a spectrometer is coupled to an optical microscope to enable both excitation and collection of Raman spectra. Additionally, the high-quality of optical microscopes makes it possible to obtain measurements with diffraction-limited spatial resolution (approximately half wavelength of the excitation laser). Spatial resolution along the optical axis can be improved by using a confocal set-up, which employs a small pinhole (less than 100 μm; in front of the detector to reject the out-of-focus photons. While certain microscopes use such pinholes, other designs use the actual detector pixels or the aperture of a collection optical fibre as a confocal pinhole. Confocal set-ups report sampling volumes as small as 1.4 fL from inside single cells [31].

The development of high power near-infrared lasers, especially 785 nm and 830 nm, was a key factor for the measurement of Raman spectra of living cells. Firstly, the high background signal associated with molecular fluorescence can be eliminated since photons do not posses the required energy to excite such transition. Secondly, near-infrared lasers induce less photodamage compared to UV or visible lasers [32-34]. This finding allowed the use of higher laser power for the excitation of Raman photons, increasing the signal strength and subsequently reducing the measurement time.

For the experiments described in this article, Raman spectra of live cells were collected using a spectrometer equipped with a high-power (∼180 mW incident on sample) laser-diode emitting at 785 nm. The laser was focused on single cells using a water-immersion objective. The focused laser spot had an elliptical shape and spatial resolution of the system was found to be Δx≅5 μm and Δy≅10 μm in the lateral directions, and Δz≅25 μm in the axial direction. Thus, the laser power density incident on cells was considerably reduced while allowing a larger volume of the cell to be probed. Raman spectra of a cell were collected in 120 seconds and several measurements were carried out from several positions of the cell to account for heterogeneity. Compared to visible lasers (488 nm and 514 nm), no morphological or spectral changes were observed for cells even after irradiations at full laser power for 40 minutes. Cells were maintained either in Petri dishes filled with phosphate buffer saline or in temperature-controlled sterile chamber, which enable monitoring of single cells exposed to toxic chemicals.

## 3. Applications

### 3.1. Raman spectra of live cells

**Figure 2.**
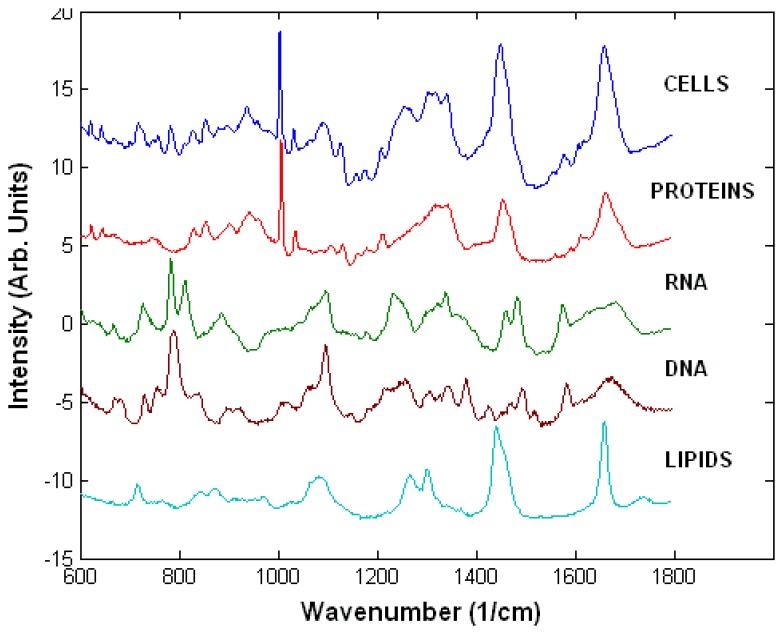
Typical Raman spectrum of a cell and of main biopolymers found in cells.

Raman spectra of live cells consist of bands corresponding to all biopolymers found in cells (Figure 2). The peak assignment of these spectra is presented in Table 1 [23]. Nucleic acids can be identified by peaks characteristic of nucleotide and sugar-phosphate backbone vibrations. The main peaks are found at 1095 cm^-1^ (phosphodioxy group PO_2_^-^), 788 cm^-1^ (C_5_′-O-P-O-C_3_′ phosphodiester bonds in DNA) and 813 cm^-1^ (C_5_′-O-P-O-C_3_′ phosphodiester bonds in RNA), 782 cm^-1^ (thymine, cytosine and uracil) and 1578 cm^-1^ (guanine and adenine). The phosphate peaks are particularly useful to determine the structure of DNA (A, B or Z forms) and also provide the main distinction between DNA and RNA.

The spectra of proteins are dominated by peaks corresponding to the Amide I (1660-1670 cm^-1^) and Amide III (1200-1300 cm^-1^) vibrations, which have been shown to be sensitive to the secondary structure of the proteins. There are additional Raman peaks corresponding mainly to amino acids containing phenyl groups, such as phenylalanine (1005 cm^-1^), tyrosine (854 cm^-1^) and tryptophan (760 cm^-1^) as well as C-H vibrations (1449 cm^-1^). While it is difficult to distinguish between specific types of proteins on the basis of their Raman spectra, the Amide I and Amide III peaks are sensitive to subtle changes in the secondary structure of proteins. The strongest Raman peaks of lipids are present at 1449 cm^-1^, 1301 cm^-1^ (C-H vibrations) and 1660 cm^-1^ (C=C stretching) and belong to vibrations of the hydrocarbon chains. Additional Raman peaks corresponding to head groups of phospholipids can also be found, such as the 719 cm^-1^ peak corresponding to the C-C-N^+^ symmetric stretching in phosphatidylcholine, a major constituent of cellular membranes. Carbohydrates can be identified and analysed reliably due to their characteristic Raman peaks of sugars, especially the C-O-C vibrations of the glycosidic bonds and sugar rings (800-1100 cm^-1^).

### 3.2. Live versus dead cells

**Figure 3.**
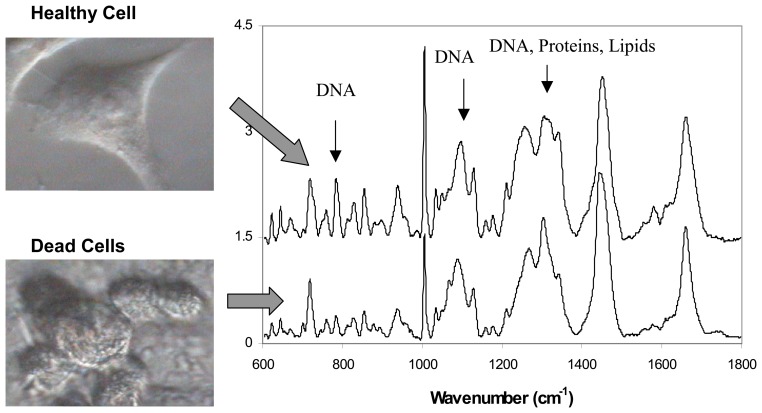
Comparison between Raman spectra of healthy and dead cells. Significant differences are observed related to DNA, proteins and lipids.

A first requirement for a biosensor is the ability to discriminate between healthy and dead cells. To test this ability, Raman spectra of healthy and dead cells were compared to identify the main spectral differences [35]. Cells were visually inspected and a small number of cells which showed fragile cell attachment were identified as dead/dying cells. Figure 3 shows micrographs of an individual healthy and dead cells. After the Raman spectra were measured, Trypan blue viability test was carried out to confirm cell death.

Comparison between measured Raman spectra of healthy live cells and dying cells revealed significant differences which were used as markers of cell viability [35] (Figure 3).

The Raman spectra of dead cells (human lung derived, A549 cell line) reflected the breakdown of phosphodiester bonds and DNA bases as indicated by the reduction of 788 cm^-1^ and 782 cm^-1^ Raman peaks by 80% and 66% respectively. In addition, the Raman peaks of proteins were found to decrease by 33% (1320 cm^-1^) and 44% (1342 cm^-1^). The sharp peak corresponding to phenylalanine (1005 cm^-1^) also decreases drastically by 45%. Establishing these spectral signatures of cell death should make it possible to use Raman spectroscopy to monitor the health of cells and for detection of toxic agents.

### 3.3. Interaction of cells with drugs

Raman spectroscopy can be used for developing cell-based biosensors for monitoring the interaction of cells with drugs. The strength of Raman spectroscopy for *in-vitro* analyses is the ability to establish the statistical effects of the drug exposure in real time on human cells of a specific phenotype that is relevant to the efficacy of the drug being tested.

**Figure 4.**
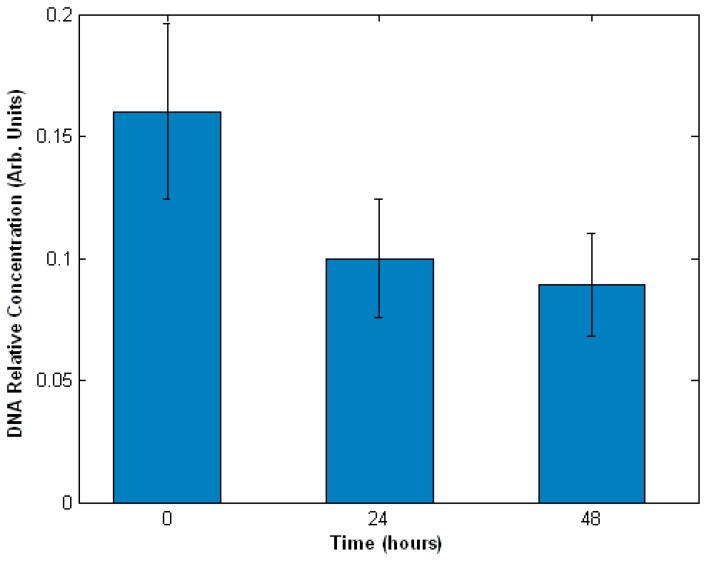
Damage to DNA caused by exposure of A549 cells to etoposide.

We investigated the ability of this technique to detect and quantify the damage induced to DNA by a genotoxic anti-cancer drug etoposide, a topoisomerase II inhibitor [36]. Type II pneumocyte-like cells (A549 cell-line) were exposed 100 μM etoposide and Raman spectra were measured at 24 and 48 hours. Least Square Fitting analysis was used to quantify DNA degradation in A549 cells. In this method, a Raman spectrum of a cell is modelled as a linear combination of Raman spectra measured from pure biopolymers. Thus, the fitting parameters can be interpreted as relative concentrations of the respective chemical species. Using this analysis method, it was found that the concentration of DNA decreased by ∼40% and ∼90% after 24 and 48 hours (Figure 4). These results were confirmed by Western blot analysis of apoptosis regulator protein p53 [36].

#### Toxicology of chemical and biological warfare

The capability of Raman micro-spectroscopy to discriminate between the cellular effects of ricin and sulphur mustard (SM), two toxic agents of bioterrorism and chemical warfare significance was investigated. Ricin, a potent and potentially lethal ribosome inactivating protein isolated from the seeds of the castor bean plant (*Ricinus communis*). Sulphur mustard was first used in War World 1 and is still a chemical warfare agent of concern. Sulphur mustard is a potent bifunctional alkylating agent that causes debilitating chemical burns to the skin, eyes and respiratory tract. Raman spectra of type II pneumocyte-like cells (A549 cell line) exposed to ricin or to sulphur mustard at various concentrations were acquired and subsequently analysed using the Linear Discriminant Analysis (LDA). In this method, linear combinations of variables are computed to determine directions in the spectral space, discriminant functions (LDs), that maximise the variance between groups and minimise the variance within groups according to Fisher's criterion. For the validation of the LDA model, the leave-one-out cross-validation was used. In this method, all spectra except one were used to build a LDA model and then to classify the left out spectrum. This method is repeated so that each spectrum is predicted once.

**Figure 5.**
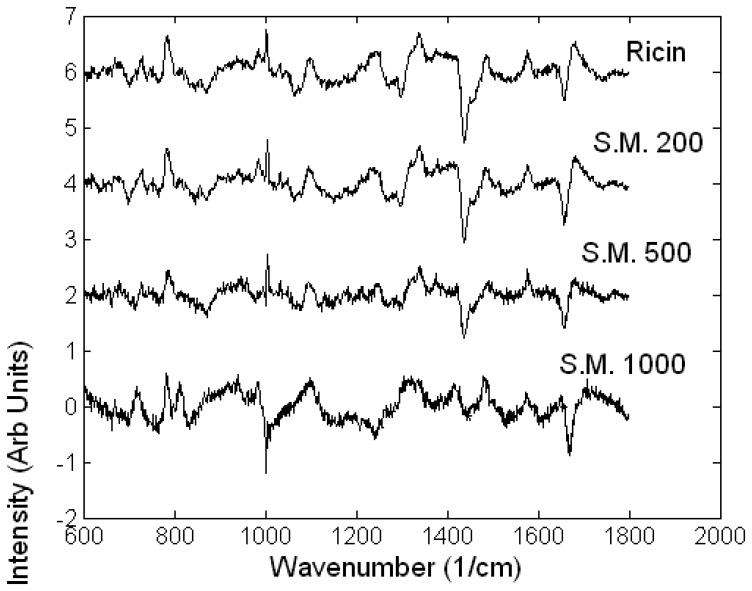
Difference between the Raman spectra of healthy A549 cells and A549 cells exposed for 24 hours to ricin (Ricin 10 nM) and sulphur mustard (S.M. at 200 μM, 500 μM and 1000 μM).

Differences between Raman spectra of healthy A549 cells and A549 cells exposed for 24 hours to ricin (10 nM) and SM (200-1000 μM) are shown in Figure 5 [37].

**Figure 6.**
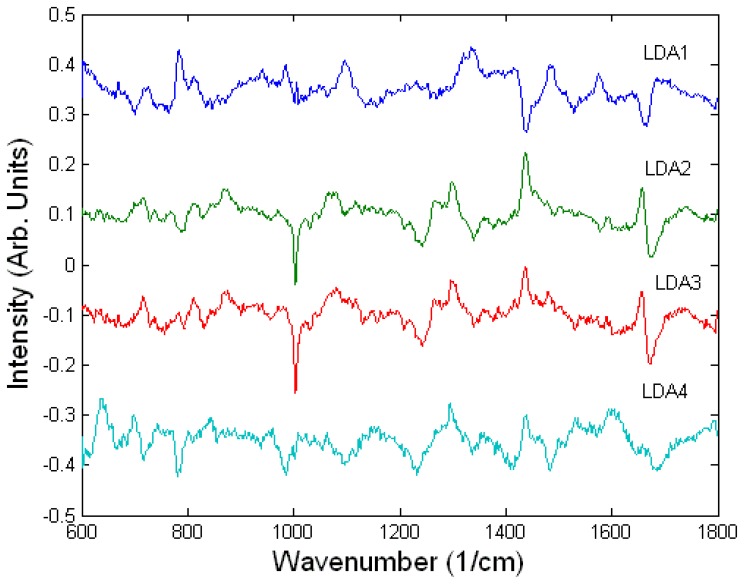
LDA loadings for the model used to discriminate between the toxicology of ricin and sulphur mustard on A549 cells

The difference Raman spectra suggest that there are similarities between the spectral changes of A549 cells treated with ricin and the lower concentrations of sulphur mustard (200-500 μM) compared to high concentration of sulphur mustard (1000 μM). Since the two cell death mechanisms, apoptosis and necrosis, involve different biochemical processes, the measured spectral changes can be used as discriminating signatures for the two toxic agents and may also be used to determine the concentration of the toxic agents. The A549 cells undergoing apoptosis showed a relative decrease in the Raman peaks corresponding to DNA (782 cm^-1^ and 788 cm^-1^, DNA) and proteins (1005 cm^-1^, HSA) and an increase in the Raman peaks corresponding to lipids (1301 cm^-1^, 1449 cm^-1^, 1660 cm^-1^). In comparison, the necrotic A549 cells treated with the highest concentration of sulphur mustard showed no apparent changes in the Raman peaks corresponding to DNA and lipids but a decrease in the peaks corresponding to RNA (782 and 813 cm^-1^, RNA), and an increase in the protein peaks (1005 cm^-1^). These conclusions are also supported by inspecting the loading of the LD2 (highest discrimination between cells treated with ricin and sulphur mustard at all concentrations) and LD3 (discrimination between the cells exposed to various concentrations of sulphur mustard). The loadings of LD2 and LD3 are very similar suggesting that in fact the discrimination is made between apoptosis and necrosis rather that the toxin used (Figure 6).

The spectral changes observed in the Raman spectra of A549 cells subjected to toxic challenges can be correlated to intracellular biochemical changes. In apoptotic cells major changes in the structure, packaging and integrity of nuclear DNA occur as do changes in cytoplasmic biochemistry and cell volume, yet cellular membranes including the plasma membrane can remain intact for prolonged periods. These features of apoptosis could therefore be responsible for the observed relative decrease in the concentrations of DNA and proteins with respect to the lipid concentration. However, the changes related to the peaks of lipids and proteins in the Raman spectra of necrotic cells are more difficult to explain.

**Figure 7.**
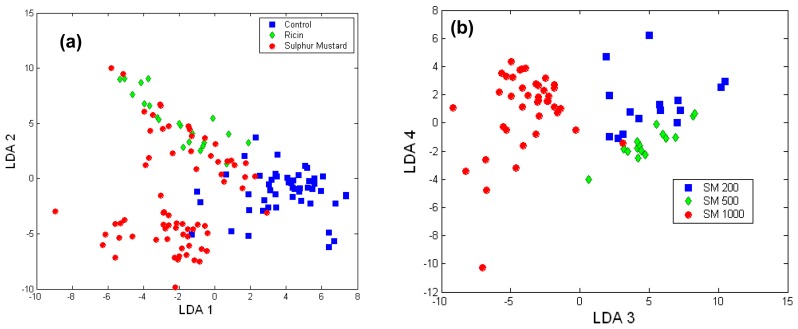
Scores of LDA model for (a) A549 cells exposed to ricin and sulphur mustard and (b) A549 cells exposed to various concentrations of sulphur mustard.

To establish the ability of Raman micro-spectroscopy to discriminate between the two toxic agents based on the biological reaction of cells, a LDA model was built. Figure 7 presents the scores of the LDA model for Raman spectra of each cell showing significant clustering according to the toxic challenge (LDA loads are presented in Figure 6). The leave-one-out cross-validation of the PCA-LDA model showed that the damaged cells can be detected with high sensitivity (98.9%) and high specificity (87.7%). High accuracy in identifying the toxic agent was also found: 88.6% for sulphur mustard and 71.4% for ricin. The prediction errors were observed mostly for the ricin treated cells and the cells exposed to the lower concentration of sulphur mustard, as they induced similar biochemical changes, as indicated by cytotoxicity assays.

The concentrations of sulphur mustard used were also identified with high accuracy: 93 % for 200 μM and 500 μM, and 100% for 1000 μM. Thus, biological Raman micro-spectroscopy and LDA analysis not only distinguishes between viable and damaged cells, but can also discriminate between toxic challenges based on the cellular biochemical and structural changes induced by these agents and the eventual mode of cell death. However, these studies examined a single time point and the possibility of examining time-dependent changes with Raman spectroscopy will more indicative for identifying and quantifying the toxic agents.

#### Monitoring single live cells

**Figure 8.**
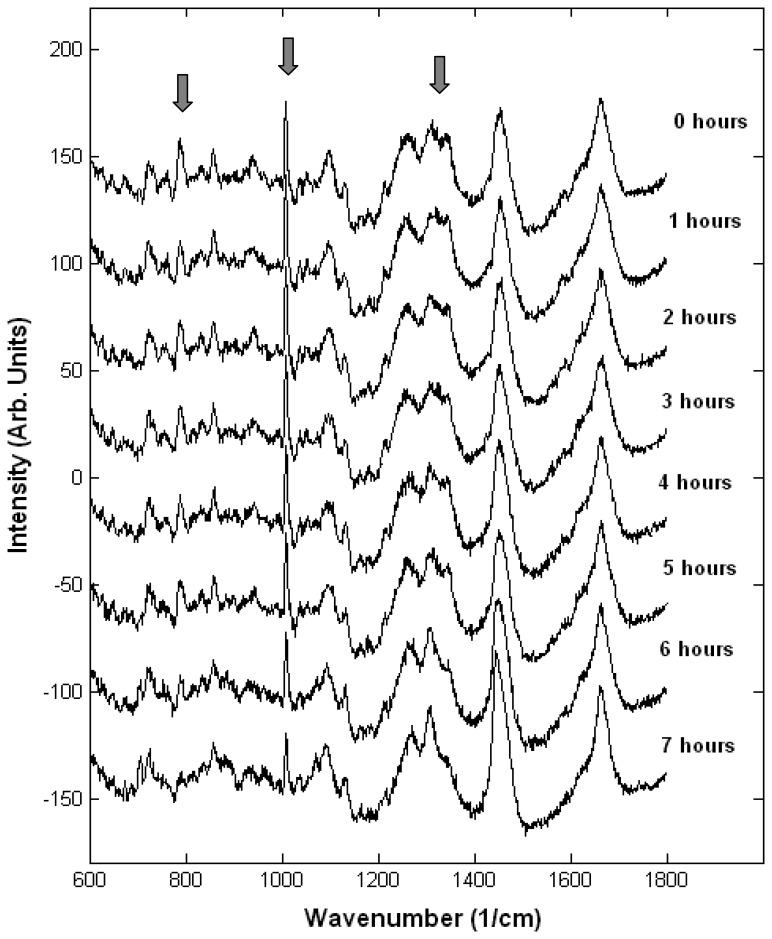
Monitoring a single A549 cell exposed to Triton ×100. The main spectral changes are indicated by the arrows.

To determine the ability of Raman micro-spectroscopy to monitor biochemical changes in cells exposed to toxic chemicals, a closed and sterile cell-chamber was developed to allow the measurements of Raman spectra of living cells in physiological conditions (constant temperature 37°C and CO_2_ independent medium). Under these conditions, we monitored the biochemical changes in individual living human lung carcinoma lung (A549) cells exposed to a toxic agent (Triton ×100) over a time period of seven hours [38]. Raman spectra measured at 60 minutes intervals from a single live cell exposed to Triton ×100 is shown in Figure 8. The measured Raman spectra indicate that major biochemical changes occur in the cell. In particular, Raman bands associated to DNA (1095 cm ^1^ phosphodioxy group PO_2_, 786 cm ^1^ phosphodiester bond C_5_′ O P O C_3_′ and nuclear bases thymine, cytosine and uracil) and phospholipids (719 cm ^1^ C C N^+^ symmetric stretching in phosphatidyl choline) show the most significant changes during the Triton ×100 treatment.

The temporal changes in the Raman peaks corresponding to the proteins in the A549 cells exposed to Triton ×100, reflect the complex biochemical changes occurring during cell death. The overall effect observed in the Raman spectra is difficult to interpret as many proteins are degraded or formed during cell death. Apart from biochemical changes related to proteins, cell death involves significant changes in the cell nucleus. The most significant change in the Raman peaks that corresponds to DNA is the decrease in the magnitude of the 786 cm ^1^ peak due mostly to the phosphodiester bond C_5_′ O P O C_3_′. The magnitude of this peak decreases as early as 60 minutes to approximately 70% of the initial value and remains stable at this value until 300 330 minutes, when a steep decrease is observed down to 10-15%. A decrease by only 40% of the initial value is found after 420 minutes. These values are similar to the values reported for dead A549 cells [35, 39].

## 4. Conclusions

The examples presented in this paper demonstrate that Raman spectroscopy is a powerful method for developing cell-based biosensors. This technique can provide information regarding cellular processes and changes in cell biochemistry following the interaction of live cells with toxic agents. There are significant spectral differences in dead cells compared to healthy cells, differences which can be used as markers for cell viability. The feasibility of this technique to identify and quantify various toxic agents was tested on two important toxic agents, ricin and sulphur mustard. The multivariate analysis model showed high sensitivity in identifying damaged cells (98.9%) and the accuracy in identifying the toxic agent were 88.6% for sulphur mustard and 71.4% for ricin. The prediction errors were observed mostly for the ricin treated cells and the cells exposed to the lower concentration of sulphur mustard, as they induced similar biochemical changes, as indicated by cytotoxicity assays.

An important feature of Raman micro-spectroscopy is the ability to monitor cellular processes over extended periods of time without damaging the cells. Live cells can be maintained in physiological conditions and their Raman spectra can be measured to determine the time-dependent biological reactions to toxic agents. This feature may be extremely useful in particular for cases in which one end point cell interrogation is not sufficient. Such cases can include toxins which induce similar cell-death mechanism but with different time-scales.

The potential of this technique for high-throughput screening of drugs and chemicals has been recognised and the feasibility established. Such studies have an important role in drug development as they may significantly reduce the time and cost of drug testing, and at the same time may help reduce the need for animal testing.

## Figures and Tables

**Table 1.** Peak assignment for Raman spectra of living cells [23].

**Peak** (cm^-1^)	**Assignment**			

	Nucleic Acids	Proteins	Lipids	Carbohydrates
1736			C=O ester	
1680-1655		Amide I	C=C str.	
1617		C=C Tyr, Trp		
1607		C=C Phe, Tyr		
1578	G, A			
1480-1420	G, A, CH def	C-H	CH def	CH def
1342	A, G	C-H		CH def
1320	G	C-H		
1301			CH2 twist	
1284-1220	T, A	Amide III	=CH bend	
1209		C-C6H5. Phe, Trp		
1176		C-H bend Tyr		
1158		C-C/C-N str.		
1128		C-N str.		C-O str
1095-1060	PO_2_^-^ str.		Chain C-C str.	C-O, C-C str
1033		C-H in-plane Phe		
1005		Sym. Ring br Phe		
980		C-C BK str. b-sheet	=CH bend	
937		C-C BK str. a-helix		C-O-C glycos.
877			C-C-N+ sym str	C-O-C ring
854		Ring br Tyr		
828	O-P-O asym.str.	Ring br. Tyr		
811	O-P-O str. RNA			
788	O-P-O str. DNA			
782	U,C,T ring br			
760		Ring breath Trp		
729	A			
717			CN+(CH3)3 str	
667	T, G			
645		C-C twist Tyr		
621		C-C twist Phe		

## References

[1] Pancrazio J.J., Whelan J.P., Borkholder D.A., Ma W., Stenger D.A. (1999). Development and applications of cell-base biosensors. Ann. Biomed. Eng..

[2] Ziegler C. (2000). Cell-based biosensors. Fresenius J. Anal. Chem..

[3] Sørensen S.J., Burmølle M., Hansen L.H. (2006). Making bio-sense of toxicity: new developments in whole-cell biosensors. Curr. Opin. Biotechnol..

[4] Lee H.J., Villaume J., Cullen D.C., Kim B.C., Gu M.B. (2003). Monitoring and classification of PAH tocixity using an immobilised bioluminescent bacteria. Biosensensors Bioelectron..

[5] Kim B.C., Park K.S., Kim S.D., Gu M.B. (2003). Evaluation of ahigh throughput toxicity biosensor and comparison with Daphnia magna bioassay. Biosensensors Bioelectron.

[6] Fernandes P.B. (1998). Technological advances in high-throughput screening. Curr. Opin. Chem. Biol..

[7] Gross G.W., Rhoades B.K., Jordan R.J. (1992). Neural networks for biochemical sensing. Sensors Actuators.

[8] Gross G.W., Azzazy H.M.E., Wu M.C., Rhodes B.K. (1995). The use of neuronal networks on microelectrode arrays as biosensors. Biosensors Bioelectron.

[9] Potter S., DeMarse T.B. (2001). A new approach to neural cell culture for long-term studies. J. Neurosci. Methods.

[10] Chiappalone M., Vato A., Tedesco M.B., Marcoli M., Davide F., Martinoia S. (2003). Networks of neurons coupled to microelectrode arrays: a neuronal sensory system for pharmacological applications. Biosensensors Bioelectron.

[11] Pancrazio J.J., Kulagina N.V., Shaffer K.M., Gray S.A., O'Shaughnessy T.J. (2004). Sensitivity of the neuronal network biosensor to environmental threats. J. Toxicol. Env. Heal. A..

[12] Natarajan A., Molnar P., Sieverdes K., Jamshidi A., Hickman J.J. (2006). Microelectrode array recordings of cardiac action potentials as a high throughput method to evaluate pesticide toxicity. Toxicol In Vitro..

[13] Liu Q., Cai H., Xu Y., Xiao L., Yang M., Wang P. (2007). Detection of heavy metal toxicity using cardiac cell-based biosensor. Biosensors Bioelectron.

[14] Yang M., Prasad S., Zhang X., Morgan A., Ozkan M., Ozkan C.S. (2003). Cellular microarrays for chemical sensing. Sensor Mater.

[15] Lorenzelli L., Margesin B., Martinoia S., Tedesco M.T., Valle M. (2003). Bioelectrochemical signal monitoring og in vitro cultured cells by means of an automated microsystem based on solid state sensor. Biosensensors Bioelectron.

[16] Rainina E.I., Efremenco E.N., Varfolomeyev S.D., Simonian A.L., Wild J.R. (1996). The development of a new biosensor based on recombinant E. coli for the direct detection of organophosphorus neurotoxins. Biosens. Bioelectron.

[17] May K.M.L., Wang Y., Bachas L.G., Anderson K.W. (2004). Development of a whole-cell-based biosensor for detecting histamine as a model toxin. Anal. Chem..

[18] Giaever I., Keese C.R. (1993). A morphological biosensor for mammalian cells. Nature.

[19] Tiruppathi C., Malik A.B., Del Vecchio P.J., Keese C.R., Giaever I. (1992). Electrical method for detection of endothelial cell shape change in real time: assessment of endothelial barrier function. Proc. Natl. Acad. Sci. USA.

[20] Mahadevan Jansen A., Richards Kortum R. (1996). Raman Spectroscopy for the detection of cancers and precancers. J. Biomed. Optics..

[21] Gremlich H.U., Yan B. (2001). Infrared and Raman Spectroscopy of Biological Materials.

[22] Thomas G.J. (1999). Raman Spectroscopy of Protein and Nucleic Acid Assemblies. Annu. Rev. Biomol. Struct..

[23] Notingher I., Hench L.L. (2006). Raman microspectroscopy: a non-invasive tool for studies of individual living cells in vitro. Expert. Rev. Med. Devices.

[24] Wood B.R., McNaughton D. (2006). Resonance Raman spectroscopy in malaria research. Expert Rev. Proteomics.

[25] Kneipp K., Kneipp H., Kneipp J. (2006). Surface-enhanced Raman scattering in local optical fields of silver and gold nanoaggregates-from single-molecule Raman spectroscopy to ultrasensitive probing in live cells. Acc. Chem. Res..

[26] Nan X., Potma E.O., Xie X.S. (2006). Nonperturbative chemical imaging of organelle transport in living cells with coherent anti-stokes Raman scattering microscopy. Biophys. J..

[27] Zhang X., Yonzon C.R., Young M.A., Stuart D.A., Van Duyne R.P. (2005). Surface-enhanced Raman spectroscopy biosensors: excitation spectroscopy for optimisation of substrates fabricated by nanosphere lithography. IEE. Proc. Nanobiotechnol.

[28] Ferraro J.R., Nakamoto K., Brown C.W. (2003). Introductory Raman Spectroscopy.

[29] Long D.A. (1977). Raman spectroscopy.

[30] Lewis I., Edwards H. (2001). Handbook of Raman Spectroscopy.

[31] Uzunbajakava N., Lenferink A., Kraan Y., Willekens B., Vrensen G., Greve J., Otto C. (2003). Non resonant Raman imaging of protein distribution in single human cells. Biopolymers.

[32] Neuman K.C., Chadd E.H., Liou G.F., Bergman K., Block S.M. (1999). Characterization of photodamage to escherichia coli in optical traps. Biophys. J..

[33] Puppels G.J., Olminkhof J.H.F., Segers-Nolten G.M.J., Otto C., de Mul F.F., Greve J. (1991). Laser irradiation and Raman spectroscopy of single living cells and chromosomes: sample degradation occurs with 514.5 nm but not with 660 nm laser light. Exp. Cell Res..

[34] Wood B.R., Caspers P., Puppels G.J., Pandiancherri S., McNaughton D. (2007). Resonance Raman spectroscopy of red blood cells using near-infrared laser excitation. Anal. Bioanal. Chem..

[35] Notingher I., Verrier S., Haque S., Polak J.M., Hench L.L. (2003). Spectroscopic study of human lung epithelial cells (A549) in culture: Living cells versus dead cells. Biopolymers.

[36] Owen C.A., Selvakumaran J., Notingher I., Jell G., Hench L.L., Stevens M.M. (2006). In vitro toxicology evaluation of pharmaceuticals using Raman micro-spectroscopy. J. Cell. Biochem..

[37] Notingher I., Green C., Dyer C., Perkins E., Hopkins N., Lindsay C., Hench L.L. (2004). 2004a Discrimination between ricin and sulphur mustard toxicity in vitro using Raman spectroscopy. J. R. Soc.:Interface.

[38] Notingher I., Selvakumaran J., Hench L.L. (2004). New Detection System for Toxic Agents Based on Continuous Spectroscopic Monitoring of Living Cells. Biosens. Bioelectron.

[39] Verrier S., Notingher I., Polak J.M., Hench L.L. (2004). In situ monitoring of cell death using Raman microspectroscopy. Biopolymers.

